# Recurrent Cutaneous Herpes Simplex Virus Infection in an Immunocompetent Young Adult: A Case Report

**DOI:** 10.7759/cureus.115329

**Published:** 2026-08-27

**Authors:** Astrid M Widjaja, Emmalee Todd, Danielle Y Baek, Stacy Schwartz, Mihir K Patel

**Affiliations:** 1 Internal Medicine, University of Maryland Medical Center, Baltimore, USA; 2 Medicine, University of Maryland Medical Center, Baltimore, USA; 3 Internal Medicine, University of Maryland School of Medicine, Baltimore, USA

**Keywords:** acylovir, cutaneous, dermatology, hsv, infectious disease, rash, shingles, vesicular, vzv

## Abstract

The herpes simplex virus (HSV) is a common cause of painful vesicular rash, which can often be mistaken for other causes of vesicular eruptions, such as herpes zoster, due to their similar physical presentation. This case report describes a woman in her 30s who presented with a recurrent vesicular rash on her left thigh. The rash had previously been diagnosed as herpes zoster. Given her young age, recurrent lesions in the same location, and absence of immunocompromising conditions, further evaluation was pursued to assess for an alternative diagnosis. The results ultimately led to a diagnosis of cutaneous HSV infection. Although the initial treatment for the rash did not differ, this case highlights the importance of differentiating between HSV and herpes zoster, as the overall evaluation, long-term management, patient counseling, and recurrence patterns differ between the two infections.

## Introduction

Herpes simplex virus (HSV) infections are among the most prevalent viral infections worldwide, with an estimated 3.75 billion people infected with HSV-1 and 491.5 million with HSV-2 [1]. These double-stranded DNA viruses establish lifelong latency following primary exposure, with the capacity for periodic reactivation throughout the host's lifetime [2,3]. In the United States, HSV-1 seroprevalence reaches approximately 48% among adults aged 14-49 years, while HSV-2 affects approximately 12% of individuals in this age group [4,5]. 

Cutaneous HSV infection follows a characteristic clinical course. Following an incubation period of 2-12 days (average of four days), symptomatic infections often present with a sensory prodrome of pain, burning, or tingling, followed by the development of erythematous macules and papules that evolve into thin-walled vesicles on an erythematous base. These vesicles subsequently pustulate, ulcerate, and crust over a period of 5-10 days in immunocompetent hosts. Primary infections are often accompanied by systemic symptoms including fever, headache, and regional lymphadenopathy in 39%-68% of cases, while recurrent infections typically present with milder, localized symptoms [2,6]. 

Cutaneous HSV infections can present with clinical features that closely mimic herpes zoster (shingles), leading to diagnostic confusion and potential delays in appropriate management. Both HSV and varicella-zoster virus (VZV, the cause of shingles) are members of the Herpesviridae family and can cause vesicular eruptions, neuralgia, and atypical skin manifestations. Although herpes zoster is common, recurrent herpes zoster is relatively uncommon, particularly in young, immunocompetent individuals, and recurrent dermatomal eruptions should prompt consideration of alternative diagnoses such as HSV. Several case reports and reviews highlight that atypical or non-vesicular presentations of HSV, particularly in the absence of classic perioral or genital lesions, are easily misdiagnosed as herpes zoster, especially in outpatient settings or where diagnosis may rely solely on clinical examination [7]. Such misdiagnosis can lead to inappropriate treatment and long-term risk assessment and management [8,9]. Here we present a case of cutaneous HSV-2 originally diagnosed as recurrent VZV and review key considerations in the diagnosis as well as implications for acute and chronic management.

## Case presentation

**Figure 1:**
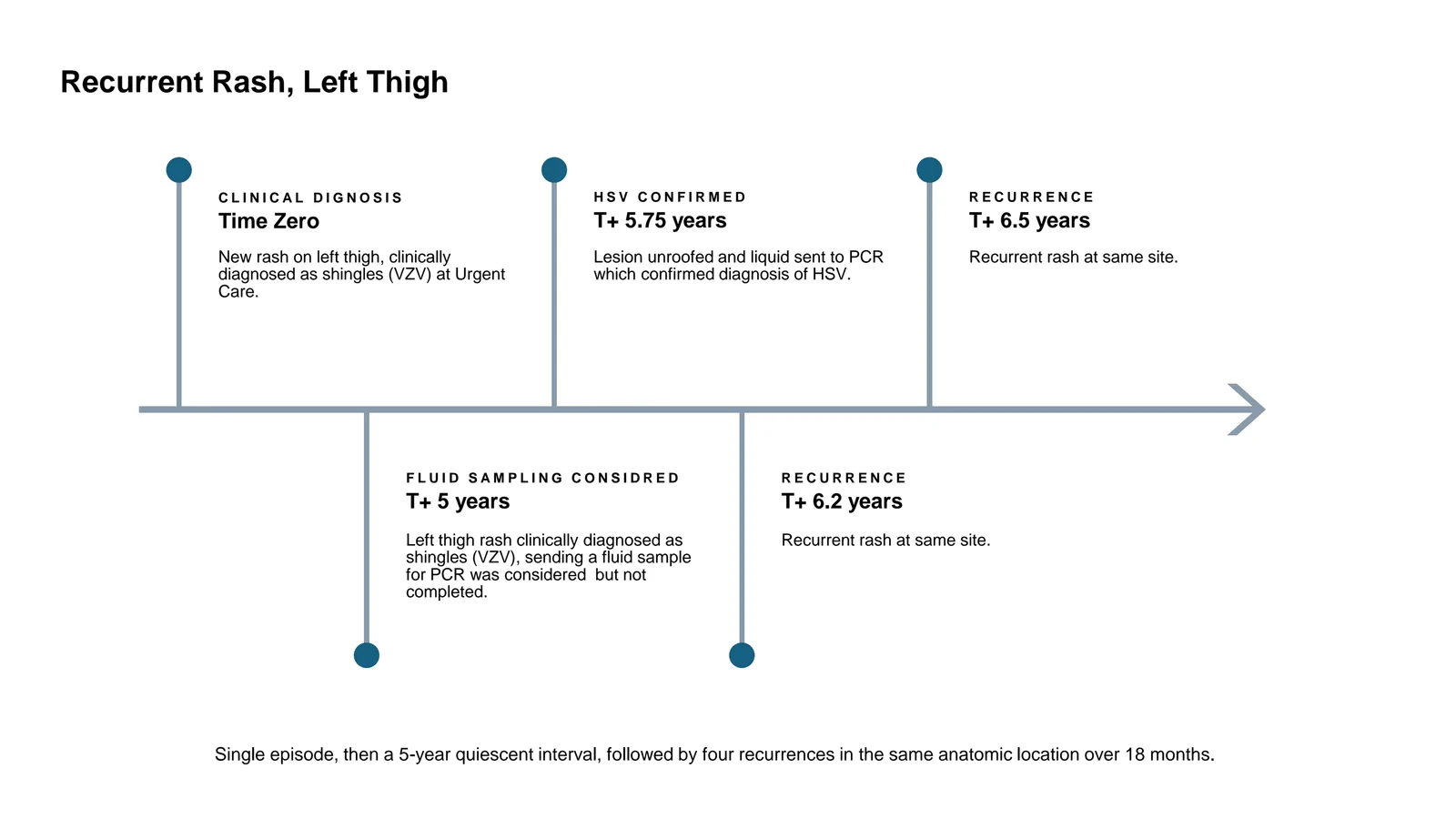
Clinical timeline of recurrent cutaneous herpes simplex virus (HSV). Time course of the recurrent vesicular rash on the left thigh. The recurrent rash continued to enlarge on the left anterior thigh and did not cross dermatomes. The diagnosis was made based on clinical findings, as laboratory testing was not performed. This image was created using Microsoft PowerPoint (Microsoft Corporation, Redmond, WA). VZV, varicella-zoster virus

A woman in her late 30s presented to an internal medicine clinic with a two-day history of a recurrent vesicular eruption that she described as a *shingles* flare. She described a blistering rash on her left anterior thigh with antecedent skin paresthesia without systemic symptoms of fever or malaise. Six years prior, she presented to urgent care with a unilateral, painful, erythematous, vesicular rash on the left anterior thigh. She was clinically diagnosed with herpes zoster and treated with a seven-day course of valacyclovir, resulting in complete resolution of her symptoms. Nine months before our evaluation, she experienced a recurrence of a similar rash in the same location and was treated with a seven-day course of famciclovir, with subsequent resolution. At that time, sampling the rash was considered but not completed. The rash consisted of grouped vesicles on an erythematous, nonindurated base without crusting. It did not cross the midline and was localized to the left L2 dermatome, with no associated lymphadenopathy. The rash had never occurred elsewhere on her body, and she denied any associated systemic symptoms during prior episodes. The patient stated that she had been told that her frequent recurrences of shingles were likely due to stress. Her medical history was notable for hyperlipidemia and prediabetes; her most recent hemoglobin A1c two months prior was 5.4% (Table 1). She denied any history of immunocompromising conditions and had never taken biologic agents nor systemic steroids. The patient’s clinical timeline is summarized in Figure 1.

**Table 1:** Recent laboratory results. The pertinent laboratory results from the patient’s most recent complete blood count (CBC), comprehensive metabolic panel (CMP), and thyroid-stimulating hormone (TSH) panel are displayed. Her laboratory results were unremarkable and did not suggest an acute diagnosis. BUN, blood urea nitrogen; eGFR, estimated glomerular filtration rate

Labs	Value	Normal values
Creatinine	1.1	0.5-1.0 mg/dL
BUN	11	7-17 mg/dL
eGFR	65	>60 mL/min/1.73 m^2^
Hemoglobin	13	11.2-15.7 g/dL
WBC	3.6	4.0-10.6 x 10^3^/uL
TSH	0.87	0.470-4.680 µIU/mL
HbA1c	5.4	<5.7%

**Figure 2:**
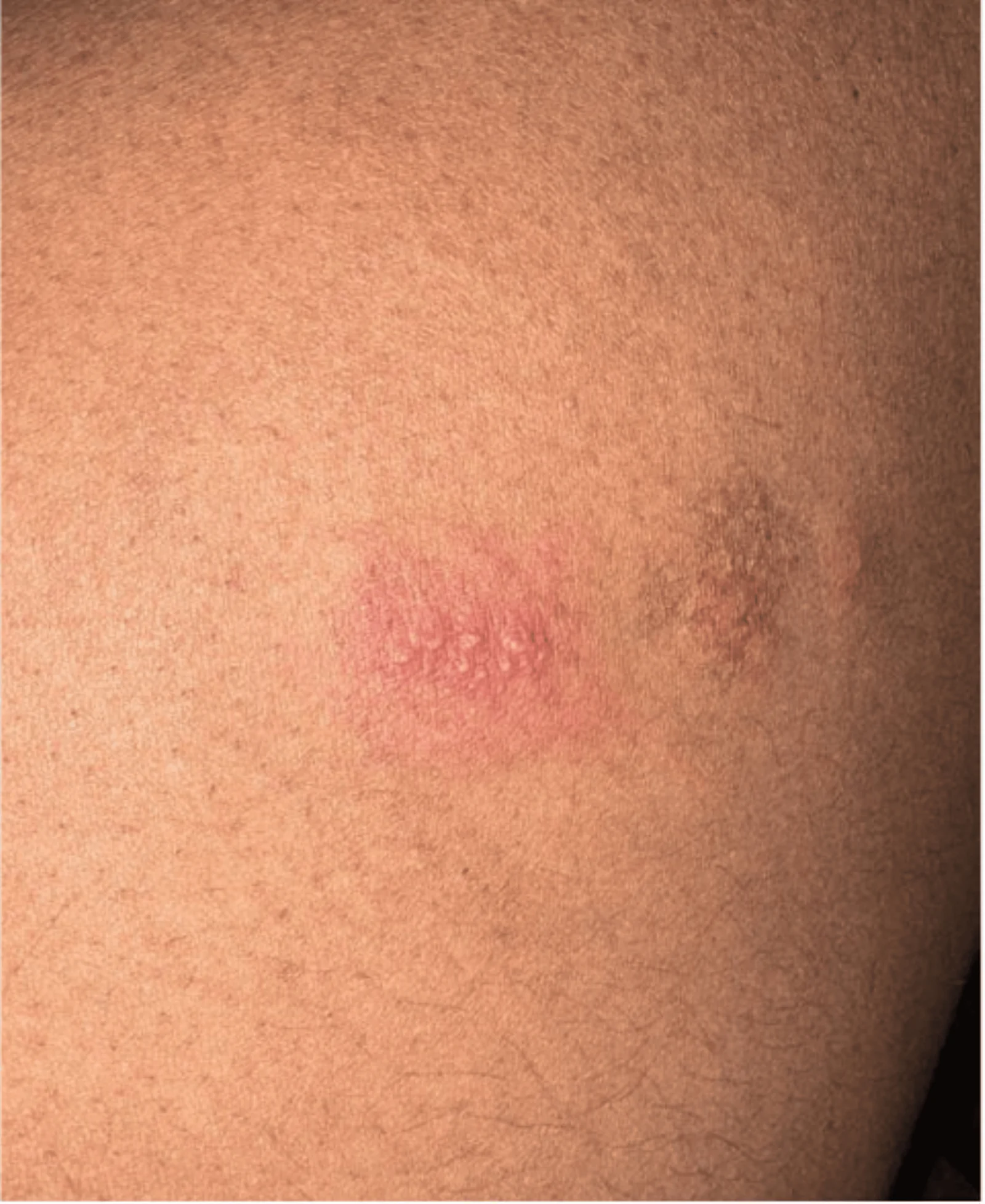
Vesicular rash on the thigh. Erythematous rash with vesicles on the left thigh.

Vital signs were within normal limits. Examination of the left proximal anterior thigh revealed an approximately 3 × 3 cm group of vesicular lesions on an erythematous base that was markedly tender to touch (Figure 2). The remainder of the physical examination was unremarkable. The patient’s frequent recurrences of *shingles* at a young age, with no history of immunosuppressive conditions or medications, were felt to warrant further investigation for either a viral etiology other than VZV, such as nonoral, nongenital HSV, or a previously unrecognized immunodeficiency. An HSV PCR sample was obtained by unroofing the largest vesicles, and the base of the unroofed vesicle was swabbed for HSV PCR. The patient was prescribed a seven-day course of valacyclovir and advised that she would be contacted with the PCR results and that, if negative for HSV, she would likely be asked to undergo further workup for causes of immunosuppression. 

The PCR was positive for HSV-2. A secure portal message was sent to the patient to inform her of the results and advise her that an additional prescription for valacyclovir had been sent to her pharmacy to be taken at the first sign of a future flare. Of note, she had no history of oral or genital herpes but had undergone STI screening at a routine gynecologic visit three years earlier, which was positive for HSV-2 IgG. She had no oral, genital, or skin lesions or symptoms at the time. She was advised to contact the clinic if she began experiencing more frequent flares so that daily prophylactic antiviral therapy could be considered. She had two more flares over the next 18 months, each four months apart, which were again successfully treated with valacyclovir.

## Discussion

Although this patient was originally diagnosed with recurrent shingles, her young age, absence of immunocompromising conditions, and repeated eruptions in the same anatomical location raised suspicion for an alternative diagnosis. The differential diagnosis of a vesicular eruption includes HSV, contact dermatitis, cellulitis, impetigo, autoimmune blistering disorders such as bullous pemphigoid and dermatitis herpetiformis, drug reactions, enteroviral infections, varicella, mpox, rickettsial infections, bacterial infections such as Bartonella henselae, and other inflammatory dermatoses. Of these possibilities, HSV remains the most clinically significant, as it can mimic herpes zoster and present in young, immunocompetent patients with recurrent lesions in the same dermatome. 

HSV exists as two distinct types, HSV-1 and HSV-2, which differ in their sites of infection and recurrence patterns [1,2]. HSV-1 is typically associated with orolabial disease, whereas HSV-2 commonly causes genital infections and is associated with more frequent recurrences. Both types of HSV can infect cutaneous or mucosal surfaces. Cutaneous HSV lesions presenting in a dermatomal distribution have been referred to as *zosteriform HSV* and closely mimic herpes zoster, contributing to diagnostic confusion [10,11]. Polymerase chain reaction (PCR) testing of vesicular lesions is the gold standard diagnostic procedure because of its superior sensitivity and specificity compared with viral culture or Tzanck smear [2]. Distinguishing HSV from VZV presents a diagnostic challenge, particularly when lesions recur in the same dermatomal location. Both viruses are members of the Herpesviridae family, present as vesicular eruptions, and establish lifelong latency within sensory ganglia after primary infection. Despite these similarities, their general recurrence patterns differ. Reactivation of VZV typically occurs years after primary infection and is most common in older adults or immunocompromised individuals. In contrast, HSV frequently reactivates and tends to recur within the same anatomical location [1,2,12].

Recurrent herpes zoster in immunocompetent adults under the age of 50 is uncommon and should prompt evaluation for an underlying immunocompromising condition or an alternative diagnosis. Most studies detailing the recurrence patterns of herpes zoster focus on patients over the age of 50, with very limited data describing recurrence in immunocompetent individuals under the age of 50 [12]. Epidemiologic data further support the rarity of recurrent herpes zoster in young, immunocompetent patients. General population studies estimate that 1.2-9.6% of individuals will experience herpes zoster recurrence within their lifetime [12]. The recurrence rate among immunocompetent, unvaccinated adults over the age of 50 has been reported as approximately 10.96 cases per 1,000 person-years [13]. The recurrence rate among a population of Australian individuals between the ages of 45 and 54 was determined to be 10.2%. However, this study may be subject to ascertainment bias [14]. Nevertheless, overall HSV recurrence occurs more frequently than herpes zoster recurrence. Data on nongenital HSV-2 recurrence are limited; however, a cohort study from 1994 reported that 21% of patients with primary genital HSV-2 infection subsequently developed nongenital recurrences [15]. Overall, the literature underscores that clinicians should remain cautious about premature diagnostic anchoring on herpes zoster and avoid overattributing recurrent dermatomal eruptions to VZV, particularly in younger, immunocompetent patients. A limitation of this case is that PCR confirmation was not performed during earlier episodes. Although the recurrent eruptions were reported to have a similar distribution and clinical appearance, the absence of virologic testing limits definitive confirmation that these prior episodes represented recurrent VZV infection. 

Inaccurate differentiation between HSV and VZV can generate patient anxiety and prevent appropriate medical and public health counseling, as cutaneous HSV carries different implications for transmission, sexual health, and pregnancy compared with VZV [8]. HSV-2 infection requires focused education on safe sexual practices, and recurrent HSV infection may involve long-term antiviral therapy, while VZV management centers on vaccination strategies and post-exposure prophylaxis, especially in vulnerable populations. Misidentification can hinder effective counseling on transmission risks, delay appropriate interventions, and ultimately affect patient outcomes and public health efforts. Although antiviral medications such as valacyclovir and acyclovir are effective treatments for both infections, the expected recurrence pattern, dosing and duration, and long-term management strategies differ [3]. Recurrent HSV infections are managed with episodic antiviral treatment at the onset of prodromal symptoms, with consideration of long-term suppressive therapy for those with frequent recurrences. In contrast, recurrent herpes zoster would prompt an evaluation for an immunocompromising condition, such as HIV, malignancy, medication-related immunosuppression, or primary immunodeficiency [12].

## Conclusions

This case highlights the importance of reconsidering a diagnosis of recurrent herpes zoster in young, immunocompetent patients. Despite previous diagnoses of herpes zoster, her age and recurrence pattern suggested HSV. PCR testing, which is highly sensitive and specific, is vital for evaluating recurrent, localized eruptions, especially in patients with an atypical history of herpes zoster. Accurate identification of HSV allows for tailored management, including counseling and treatment, and improves patient understanding of the disease course. This case emphasizes the importance of maintaining a broad differential diagnosis for vesicular eruptions, recognizing differences in recurrence patterns to aid diagnosis, and performing diagnostic testing of vesicular fluid when the clinical course deviates from expected VZV patterns.
